# Macroprolactinemia and Empty Sella Syndrome

**DOI:** 10.11604/pamj.2017.27.278.11361

**Published:** 2017-08-14

**Authors:** Ach Taieb, Kacem Njah Maha, Yosra Hasni El Abed, Amel Maaroufi Beizig, Molka Chaieb Chadli, Koussay Ach

**Affiliations:** 1 Endocrinology and Diabetes Department, University Hospital Farhat Hached Sousse, Tunisia

**Keywords:** Hyperprolactinemia, macroprolactinemia, Empty Sella Syndrome, pregnancy

## Abstract

Macroprolactinemia is a polymeric form of prolactin-release, causing mildly symptomatic clinical pictures. The former can be isolated or associated with other causes of hyperprolactinemia. The association with an empty sella syndrome is rare. We report a case of a female patient discovered with this association. It’s about a female patient 47 years old, followed up since the age of 31 years for bilateral galactorrhea and a spaniomenorrhea. There has been no associated drug intake. Her exploration has showed a serum prolactin level of 635 mIU/L. Thyroid test results were normal T4 = 10,2ng/L and TSH = 1.76 mIU/L. A brain scan has showed an empty sella turcica. Despite the unchanged levels of prolactinemia, the evolution under dopaminergic 5 mg /D has been marked by the occurrence of a pregnancy with persistent moderate hyperprolactinemia in the postpartum. Chromatography has showed a predominance of the macroprolactin form with: Prolactin monomer at 4.8%, Big Prolactin at 5% and Big Big Prolactin at 83%, thus stopping bromocriptine. Our observation suggests that macroprolactinemia can be associated with conventional etiologies of moderate hyperprolactinemia as the empty sella syndrome. Its detection would prevent the use of dopaminergic therapy which seems not useful.

## Introduction

Prolactin (PRL) exists in the blood under many forms: monomeric (60 to 85%), polymeric and PRL-protein complexes of higher molecular weight [1]. Macroprolactinemia is a polymeric form of prolactin-release, causing mildly symptomatic clinical pictures [2]. The former can be isolated or associated with other causes of hyperprolactinemia [3]. We report the case of a macroprolactinemia being discovered in a patient with an empty sella Syndrome.

## Patient and observation

It consists in a 47 years old female patient, who has been followed up since the age of 31 years for bilateral galactorrhea and a spaniomenorrhea. There has been no associated drug intake. Her exploration has showed a serum prolactin level of 635 mIU /L. Thyroid test results were normal T4 = 10,2ng/L and TSH = 1.76 mIU/L. A brain scan has showed an empty sella turcica. Despite the unchanged levels of prolactinemia, the evolution under dopaminergic 5 mg /D has been marked by the occurrence of a pregnancy with persistent moderate hyperprolactinemia in the postpartum. The patient has had menopause at the age of 47, with elevated gonadotrophins FSH = 78 mIU/mL, LH = 33 mIU/mL and estradiol: 35 pg/mL. Chromatography has showed a predominance of the macroprolactin form with: Prolactin monomer at 4.8%, Big Prolactin at 5% and Big Big Prolactin at 83%. After the discovery of the macroprolactinemia, we decided to stop bromocriuptin treatment. The clinical evolution of the patient is favorable without drugs intake.

**Abbreviations**: PRL: Prolactin; FSH: Follicle-stimulating Hormone; LH: luteinizing hormone.

## Discussion

We report a case of a patient with an association of empty sella Syndrome and macroprolactinemia. Macroprolactin is a non-bioactive prolactin isoform usually composed of a prolactin monomer and an IgG molecule having a prolonged clearance rate similar to that of immunoglobulins. The clinical picture may be completely asymptomatic [2] or mildly symptomatic in 22-46% of cases as in our observation. [3]. The big big prolactin isoform, is considered idiopathic and poorly symptomatic. Furthermore, the macroprolactinemia has been raised in our perception ahead of the non-functional character of hyperprolactinemia evidenced by the occurrence of pregnancy and after the menopause, although hyperprolactinemia has persisted which seems insensitive to bromocriptine [4], suggesting its non-functional character. Anderson AN and Al have as well evoked a case of a female patient, in whom a pregnancy occurred [5], with macroprolactinemia. The frequency of macroprolactinemia in pregnancy has been assessed by Hattori N. et al; over 109 pregnant women, 3 (2.9%) of them have had a macroprolactinemia [6]. Several dosing methods serve to diagnose macroprolactinemia [7]. Its detection with most of the available immunoassays in the same way as the monomeric form of PRL may result in a false diagnosis of hyperprolactinemia. In our case, the diagnosis has been confirmed by Gel permeation chromatography, which remains the method of choice for its detection [7, 8]. The high molecular-weight forms are predominant, despite their reduced biological activity, so they lead to overestimation of prolactinemia measured by classical immunoassays.

Macroprolactinemia is a common cause of hyperprolactinemia [9], but its research is not systematic [4]. According to two studies on 1225 [3] and 2089 [10] blood samples, macroprolactinemia frequencies have been estimated by 26% and 22% respectively. In another study realized by Alfonso A. and al on 40 patients with hyperprolactinemia, 18 (45%) have had macroprolactinemia [11]. The big-big prolactin was considered for a long time as a rather rare form of hyperprolactinemia. Nevertheless it seems to be much more present than it was initially studied. Macroprolactinemia is usually isolated [12], constituting the diagnosis of exclusion after a negative etiological investigation [2, 9]. The etiological research requires clinical, biological and radiological investigations including MRI [13], which objectified the empty sella in our patient. Although macroprolactin is commonly found in patients with hyperprolactinemia, neither symptoms nor MRI findings are useful in predicting its presence. The particularity of our case is that, it is associated with a common cause of hyperprolactinemia which is the empty sella syndrome [14]. This association has not been described; however associations with a prolactinoma are possible. Mounier et al have conducted a study on patients with macroprolactinemia, with whom we have discovered a micro-pituitary adenoma associated [15].

Macroprolactinemia reported during the Prolactinoma can reach up to 76% of the secreted prolactin. It is in fact composed of the complex monomer prolactin and circulating immunoglobulin G [16]. This fixation suggests that big big PRL has a lower receptor affinity due to the interference of the immunoglobulin and may account for the lack of clinical and biological effect upon the patient. Its high molecular weight may also reduce its access to target organs and be part to explain the poor clinical symptoms. The discovery of a macroprolactinemia may have an important clinical and therapeutic impact [1, 17], formerly neglected [9], avoiding the dopaminergic treatment led on our patient without interest for many years, as cited Vaishya and al. [4]. Several studies recommend systematic prolactin test [4, 9, 12, 18]. As these hyperprolactinemias are not pathological, a simple detection test using polyethylene glycol has been validated to avoid unnecessary investigations. Even if the clinical significance of macro-prolactinemia remains uncertain, the long-term assessment without treatment seems favorable [19]. There is a need to explore the diagnosis and pathophysiology of macroprolactinemia for improving patient care. A valid diagnostic method must be available in African centers to avoid unnecessary hormonal or radiological investigations and excessive treatments, exposing patients to several side effects.

## Conclusion

Our observation suggests that macroprolactinemia can be associated with conventional etiologies of moderate hyperprolactinemia as the empty sella syndrome. Its detection would prevent the use of dopaminergic therapy and excessive investigations.

## Competing interests

The authors declare no competing interest.
